# Measurement of the vascular pedicle width predicts fluid repletion: a cross-sectional comparison with inferior vena cava ultrasound and lung comets

**DOI:** 10.1186/s40560-015-0121-4

**Published:** 2015-12-22

**Authors:** Nawal Salahuddin, Iqbal Hussain, Hakam Alsaidi, Quratulain Shaikh, Mini Joseph, Hassan Hawa, Khalid Maghrabi

**Affiliations:** Adult Critical Care Medicine, King Faisal Specialist Hospital and Research Centre, Riyadh, Saudi Arabia; Department of Nursing, King Faisal Specialist Hospital and Research Centre, Riyadh, Saudi Arabia

**Keywords:** Vascular pedicle width, Inferior vena cava, Ultrasound, Fluid assessment

## Abstract

**Background:**

Determination of a patient’s volume status remains challenging. Ultrasound assessments of the inferior vena cava and lung parenchyma have been shown to reflect fluid status when compared to the more traditional static and dynamic methods. Yet, resource-limited intensive care units (ICUs) may still not have access to bedside ultrasound. The vascular pedicle width (VPW) measured on chest radiographs remains underutilized for fluid assessment. In this study, we aimed to determine the correlation between ultrasound assessment and vascular pedicle width and to identify a discriminant value that predicted a fluid replete state.

**Methods:**

Eighty-four data points of simultaneous VPW and inferior vena cava measurements were collected on mechanically ventilated patients. VPW measurements were compared with lung comet scores, fluid balance, and a composite variable of inferior vena cava diameter greater than or equal to 2 cm and variability less than 15 %.

**Results:**

A VPW of 64 mm accurately predicted fluid repletion with a positive predictive value equal to 88.5 % and an area under the curve (AUC) of 0.843, 95 % CI 0.75–0.93, *p* < 0.001. VPW closely correlated with inferior vena cava diameter (Pearson’s *r* = 0.64, *p* = <0.001). Poor correlations were observed between VPW and lung comet score, Pearson’s *r* = 0.12, *p* = 0.26, fluid balance, Pearson’s *r* = 0.3, *p* = 0.058, and beta natriuretic peptide, Pearson’s *r* = 0.12, *p* = 0.26.

**Conclusions:**

This study shows a high predictive ability of the VPW for fluid repletion, as compared to an accepted method of volume assessment. Given the relationship of fluid overload and mortality, these results may assist fluid resuscitation in resource-limited intensive care units.

## Background

Intravenous boluses of fluids are traditionally used to resuscitate hemodynamic instability, acute oliguria, and hyperlactatemia in the intensive care unit (ICU). However, overzealous fluid administration leads to organ congestion, organ failure, and an increased risk of death [1–8]. Currently, a variety of tools, such as central venous and pulmonary capillary wedge pressures, transpulmonary thermodilution, echocardiography and ultrasound of the inferior vena cava (IVC) and lung parenchyma are commonly used to gain information about volume status and guide fluid therapy. Bedside ultrasound to measure IVC diameter, variability with respiration [9–21], and extravascular lung water as determined by the presence of B lines or lung comets [22–29] have been shown to closely correlate with the more traditional methods of fluid assessment, such as central venous pressure, stroke volume, and pulse pressure variation. The reliability and ease of measurement of IVC and lung parenchyma ultrasound has led to widespread adoption by ICU physicians to assist in bedside assessment of volume status.

Yet, not all physicians have access to these tools; ICUs in resource-limited settings have to function without many of these facilities [30–32] and may be one of the factors contributing to inability to implement resuscitation guidelines [33] and higher severity-adjusted case-fatality rates [34, 35]. Nevertheless, the basic principles of fluid management can still be practiced if physicians can use the tools available to them to assess volume status.

Chest radiographs are normally available to most physicians. The vascular pedicle width (VPW) as seen on chest radiographs represents the mediastinal silhouette of the central vessels. It is easily measured and has been shown to correlate well with invasive hemodynamic measurements [36–38]. We therefore carried out this prospective, cross-sectional study to identify vascular pedicle width measurements that could discriminate fluid repletion as defined by inferior vena cava ultrasound measurements and fluid overload as defined by lung water on ultrasound.

## Methods

In a prospective, cross-sectional design, consecutive adult patients on controlled mechanical ventilation admitted to the surgical and medical ICUs were included. Patients with valvular heart disease, congestive heart failure, pulmonary hypertension, pericardial disease, and a history of thoracic or cardiac surgery, requiring positive end-expiratory pressures (PEEP) higher than 5 cm water, and pregnancy were excluded. Simultaneous supine, standardized chest radiographs, and ultrasound assessments of the IVC and lung parenchyma were done. For all patients included in the study, conventional portable, supine, anteroposterior chest radiographs were obtained on each patient within an hour of the ultrasound measurements. The radiographic technique involved a 40-inch source-image distance (SID), 60–70 kV peaks, and a typical 3- to 6-mA exposure adjusted to patient body habitus. Each radiograph was processed in a standard rapid processor with a processor time of 45 s. We attempted to adjust for confounding due to variation in chest radiograph technique by limiting the inclusion to very strictly standardized films as regards patient positioning, angle, ventilatory parameters, exposure, film distance, and the quality of film.

To eliminate interobserver variability, a single investigator (IH), with training and experience in critical care ultrasound, carried out all the ultrasound examinations. All VPW measurements were obtained by an investigator (NS) blinded to clinical and ultrasound data.

The study protocol was approved by the institutional research ethics committee (RAC approval no. 2141039), and the study was performed in accordance with the ethical standards laid down in the 1964 Declaration of Helsinki and its later amendments. Informed consent to participate and consent to publish was obtained from the attendant family or designated patient representatives and documented in the medical record.

### Measurement of the inferior vena cava diameter, and respiratory variation by ultrasound

Ultrasonographic assessment of the IVC was carried out using a transthoracic, subcostal approach. The transducer was positioned just below the xiphisternum 1–2 cm to the right of the midline, with the marker dot pointing towards the sternal notch. After obtaining a two-dimensional image of the IVC entering the right atrium and verifying that the IVC visualization was not lost during movements of respiration, an M-mode line was placed through the IVC 1 cm caudal from its junction with the hepatic vein and an M-mode tracing obtained. This placement ensures that the intrathoracic IVC is not measured during any part of the respiratory cycle. The M-mode tracing was recorded through three to four respiratory cycles, the image frozen, and using calipers, the maximum and minimum diameters of the IVC tracing measured. IVC respiratory variation was quantified by measuring the percent difference between the maximum and minimum diameters on the M-mode tracing.

### Lung ultrasound and quantification of B lines: lung comet score

Bilateral lung comet score was obtained by scanning eight anterolateral quadrants with the probe longitudinally applied perpendicular to the wall. Each hemithorax was divided in four areas: two anterior areas and two lateral areas. The anterior chest wall (zone 1) was delineated from the parasternal to the anterior axillary line and was divided into upper and lower halves, from the clavicle to the third intercostal space and from the third to the diaphragm. The lateral area (zone 2) was delineated from the anterior to the posterior axillary line and was divided into upper and basal halves. The sum of lung comets (B lines) found on each scanning site (0: absence; 1: B3 lines, multiple B lines 3 mm apart; 2: B7 lines, multiple B lines 7 mm apart) yields a score from 0 to 16 (please refer to Table 1) [23].

**Table 1:** Representative calculation of lung comet score

	Ultrasound finding	Score
Quadrant 1, right	No B lines	0
Quadrant 2, right	B3 lines	1
Quadrant 3, right	B7 lines	2
Quadrant 4, right	B3 lines	1
Quadrant 1, left	B7 lines	2
Quadrant 2, eft	B3 lines	1
Quadrant 3, left	B7 lines	2
Quadrant 4, left	B3 lines	1
Lung comet score/maximum possible score		10/16

No B lines, score = 0; B3 lines, score = 1; B7 lines, score = 2

### Vascular pedicle width measurement

Vascular pedicle width was measured on standardized, portable chest radiographs obtained in the supine position. Ultrasound assessments were done within 1 h period of the chest radiographs. To eliminate interobserver variation, a senior pulmonary and critical care consultant (NS) who was blinded to all other patient details measured the VPW. The vascular pedicle width was measured by dropping a perpendicular line from the point at which the left subclavian artery exits the aortic arch and measuring across to the point at which the superior vena cava crosses the right main-stem bronchus. In patients with an indistinct right border of the pedicle, the measurement was taken from the vertical, lateral border of the superior vena cava [39]. *Fluid Repletion* was defined as IVC diameter greater than or equal to 2 cm and or variation in IVC diameter less than 15 % during the respiratory cycle.

### Statistical analysis

VPW and IVC diameter, lung comet score, and 24-h fluid balance were separately compared using scatter plots with regression equations. R-values were determined using the Pearson coefficient. Multivariate linear regression analysis was utilized to determine the effects of lung comet score, fluid balance, and IVC diameter on VPW. Standardized coefficients were obtained to compare the relative effects of each variable on the VPW. Receiver operating characteristic (ROC) curves were utilized to determine the optimal cutoff value of VPW that identified *fluid repletion*, as defined by a composite variable of IVC diameter ≥2 cm and IVC variability ≤15 %. Specificity, sensitivity, and predictive values were calculated. A Bland-Altman plot was generated to check for possible bias. Analysis was performed and graphs were generated using SPSS (IBM SPSS version 22.0, Chicago, IL) software. Two-sided *p* values ≤0.05 determined statistical significance.

## Results

Eighty-four data points were collected on forty-three patients. Mean age was 54.7 ± 20 years, and 50 % patients were female. Comorbid illnesses included cirrhosis, 21 % (9 patients), malignancy, 37 % (16 patients), and acute renal failure, 65 % (28 patients). Admission diagnoses ranged from scheduled postoperative, 63 % (27 patients), to severe sepsis or shock, 44 % (19 patients). All patients were intubated and mechanically ventilated at time of data collection. Mean APACHE II and SAPS II scores were 22.4 ± 9 and 33.4 ± 22, respectively. ICU and 28-day survival rates were 86 and 79 % (Table 2).

**Table 2:** Clinical characteristics

	*n* = 43
Demographics	
Age years	54.7 ± 20
Female gender	42 (50 %)
APACHE II score	22.4 ± 9
SAPS II score	33.4 ± 22
Comorbid conditions	
Malignancy	16 (37 %)
End-stage liver disease	9 (21 %)
Acute renal failure	28 (65 %)
Diabetes	10 (23 %)
Admitting diagnosis	
Postoperative^a^	27 (63 %)
Severe sepsis/shock	19 (44 %)
Neurologic failure^b^	13 (30 %)
GI bleeding/hepatic encephalopathy	6 (14 %)
COPD exacerbation	4 (9 %)
Vasopressors	23 (53 %)
Mean arterial pressure (mmHg)	79 ± 10.3
pBNP	2743 (1800, 9253)
Serum creatinine (μM/l)	105 ± 66
Serum Lactate (μM/l)	2.2 (IQR 1.6)
S_C_VO_2_	65 ± 23.8
Net positive fluid balance	+1145 ml (250, 4527)
Hourly urine output (ml/kg/h)	0.8 (0, 218)
Central venous pressure (cmH_2_O)	8 ± 1.7
Mechanical ventilation	43 (100 %)
Peak pressure (cmH_2_0)	29 ± 4.1
PaO_2_ (mmHg)	95.4 ± 23.3
FiO_2_	0.41 ± 0.08
PaO_2_/FiO_2_ ratio	235 ± 66
Tidal volume (ml)	388 ± 78.3
Outcome	
ICU survival	37 (86 %)
28-day survival	34 (79 %)

Data is reported as means (± SD) or medians (IQR) for skewed distributions or as proportions

*APACHE II* Acute Physiology and Chronic Health Evaluation II, *SAPS II* Simplified Acute Physiology Score II, *pBNP* pro-beta natriuretic peptide

^a^Postoperative cases include solid organ transplantation, major abdominal surgery, orthopedic surgery, and head and neck surgery

^b^Includes intracranial bleeds, stroke, infections

Mean VPW was significantly different in the group with IVC diameter <2 cm compared to the group with IVC diameter ≥2 cm; 63.4 ± 12.6, 80.3 ± 11.1, *p* <0.001. Similarly, mean VPW was significantly lower in the group with IVC variability ≥15 % compared to <15 %; 57 ± 9.7, 74.1 ± 13, *p* < 0.001. No significant differences were observed between mean VPWs of patients with <8≥ lung comet scores, *p* = 0.97.

### Vascular pedicle width correlations with IVC ultrasound

**Fig. 1:**
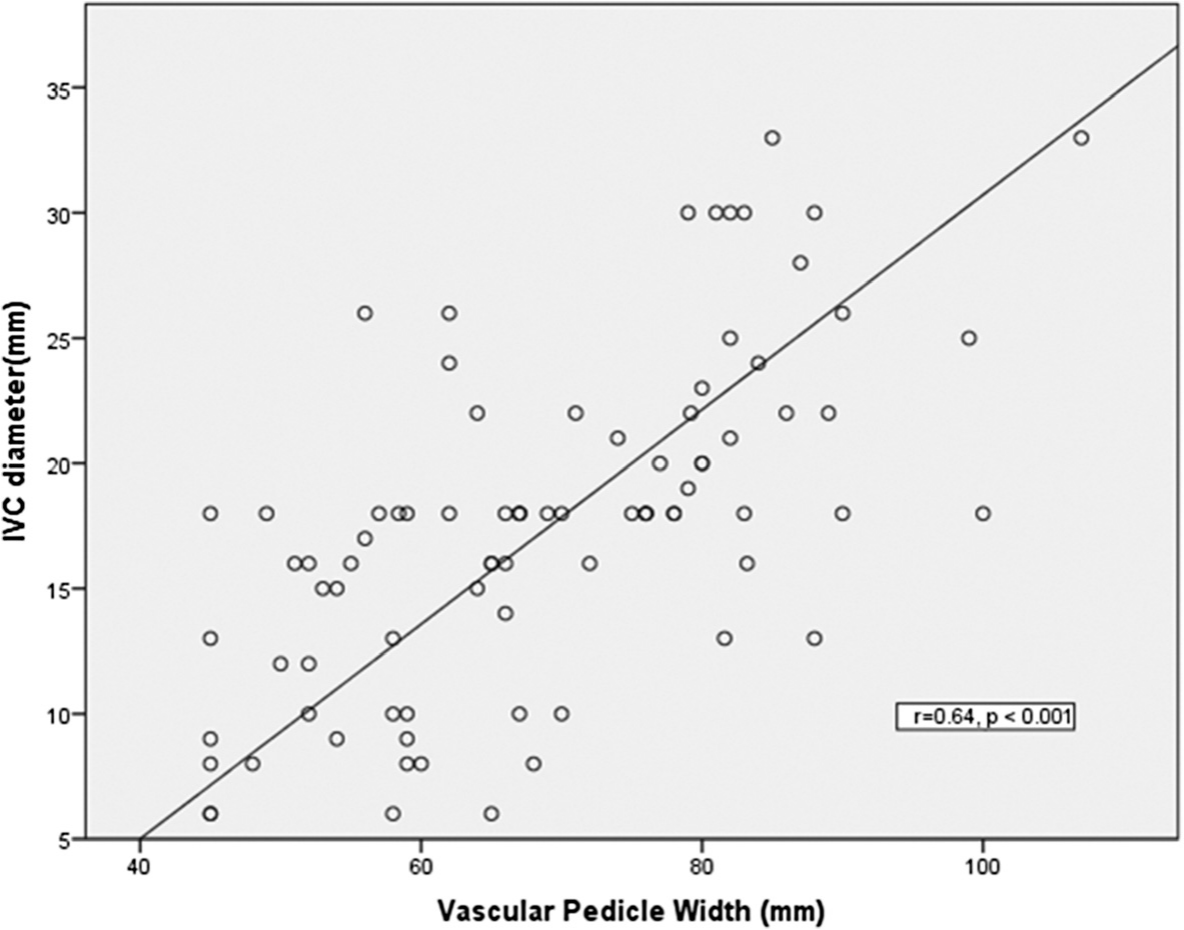
Correlation between inferior vena cava diameter and vascular pedicle width. Pearson’s correlation coefficient *r* = 0.64, *p* = <0.001

The VPW closely correlated with IVC diameter (Pearson’s *r* = 0.64, *p* = <0.001) measured on ultrasound (Fig. 1). On multivariate linear regression, standardized coefficients demonstrated that a 0.28 (beta)-mm increase in VPW corresponds to an increase in the mean IVC diameter by 1 mm (Table 3).

**Table 3:** Results of multivariate linear regression demonstrating associations between inferior vena cava diameter, lung comet score, net fluid balance, and vascular pedicle width

	Unstandardized coefficients B Std. error		Standardized coefficients	95 % CI for B	*p* value
IVC diameter	0.285	0.076	0.524	0.13, 0.44	0.001
Net fluid balance	0.002	0.001	0.181	0.001.0.003	0.20
Lung comet score	0.067	0.163	0.056	−0.02, 0.39	0.6

**Fig. 2:**
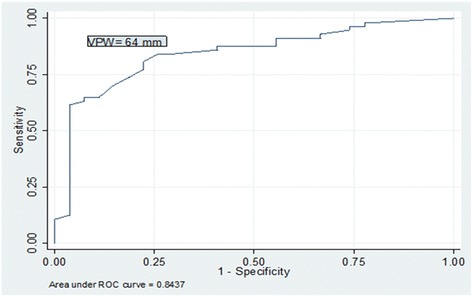
Receiver operating characteristic curve of the vascular pedicle width to identify fluid repletion shows excellent diagnostic ability at an optimal VPW cutoff of 64 mm with area under the curve (AUC) = 0.843, 95 % CI 0.75–0.93, *p* < 0.001)

A receiver operating characteristic curve was calculated to demonstrate the ability of VPW to discriminate *fluid repletion* as compared to a composite variable of IVC diameter ≥2 cm and IVC variability ≤15 %). A VPW value of 64 mm had an 81 % sensitivity, 78 % specificity for identifying fluid repletion with an area under the curve (AUC) = 0.843, 95 % CI 0.75–0.93, *p* < 0.001 (Fig. 2). This VPW value had a correct classification rate = 79.6 %, a positive predictive value = 88.5 % and a negative predictive value = 66 % to identify a *fluid replete* state.

### Vascular pedicle width correlations with lung comet score, fluid balance, and pro-beta natriuretic protein

**Fig. 3:**
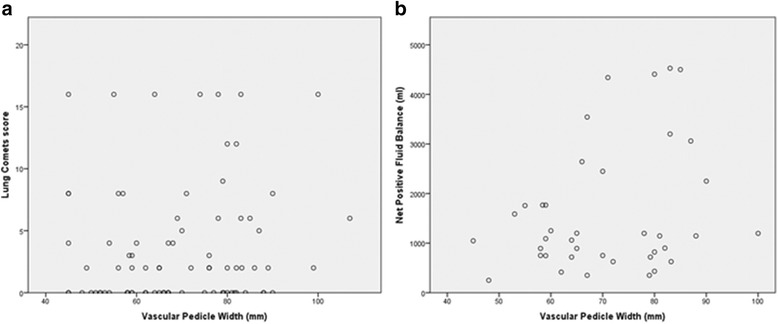
Correlations between vascular pedicle width and lung comet score (**a**) and vascular pedicle width and net fluid balance (**b**)

No significant correlation was observed between VPW and lung comet score, Pearson’s *r* = 0.12, *p* = 0.26, VPW and fluid balance, Pearson’s *r* = 0.3, *p* = 0.058, or VPW and pro-beta natriuretic protein (pBNP), Pearson’s *r* = 0.12, *p* = 0.26 (data not shown) (Fig. 3).

**Fig. 4:**
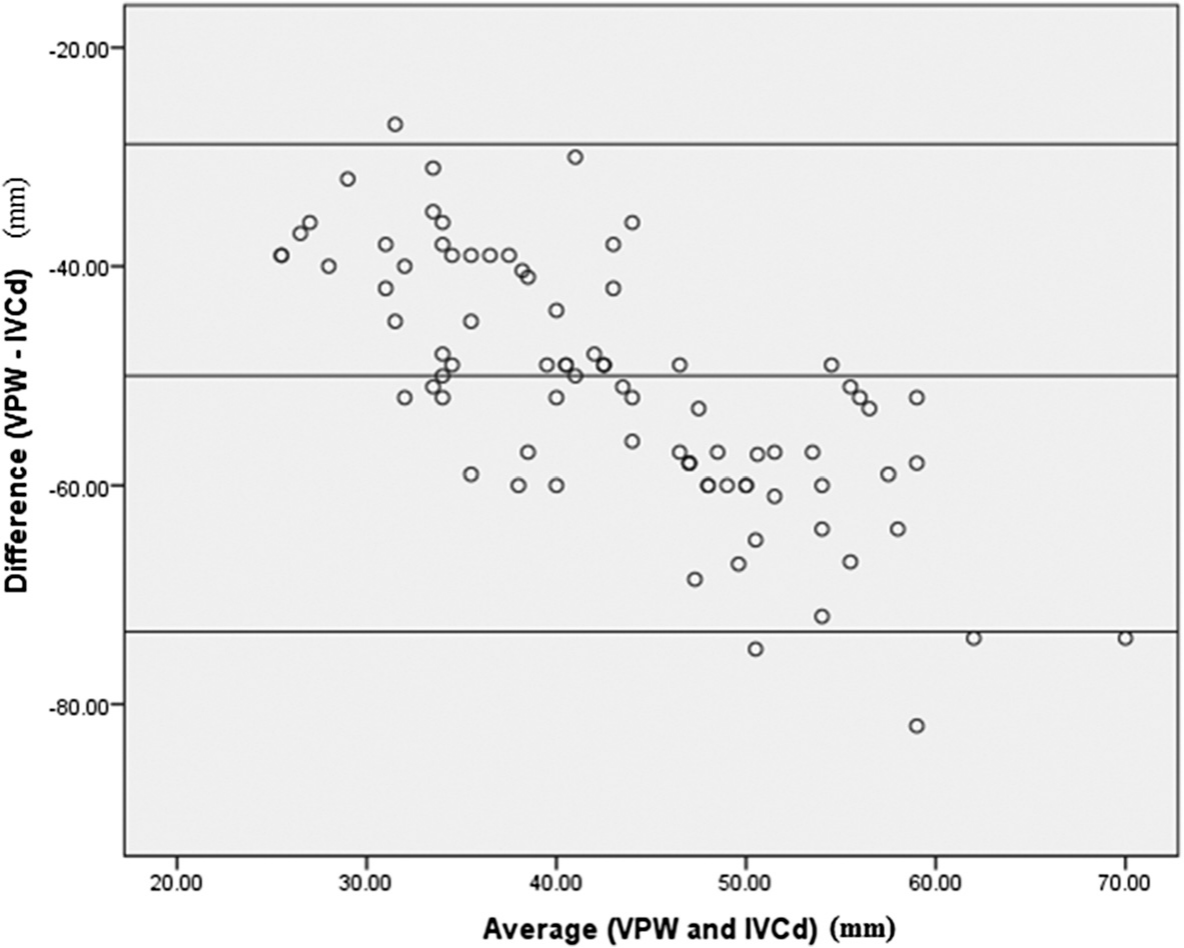
Bland-Altman plot comparing the difference (VPW-IVC diameter) with the average (of VPW and IVC diameter)

A Bland-Altman plot was constructed to assess for potential bias by comparing the VPW and IVC diameter (Fig. 4). A bias of 51.1 (mean difference of VPW − IVC diameter) was observed. Additionally, the difference and average were not independent, suggesting that in patients with low fluid status, the VPW was relatively higher than the IVC diameter and the converse was true with increasing fluid status.

## Discussion

In this study, we were able to demonstrate that a VPW cutoff value of 64 mm correctly identifies a “full” inferior vena cava and thereby fluid repletion in mechanically ventilated patients. Our comparison of the VPW against the gold standard of inferior vena cava diameter greater than 2 cm and loss of variation in diameter with the respiratory cycle will allow physicians in settings with no access to ultrasound to make a confident indirect assessment of fluid status. We found no relationship between extravascular lung water (as measured by lung comet score) and VPW, possibly since the development of pulmonary edema is not as simplistic and is governed by factors independent of central hydrostatic pressures, such as increased vascular permeability.

Making an accurate assessment of fluid status remains a daily challenge for every practicing intensive care physician. Though a myriad of tools have become available, accessibility is varied. At the same time, volume assessment is even more important because of increasing data identifying the risks associated with both liberal fluid administration [1–8, 40] and the real risks of underhydrating a dehydrated patient with a compromised microcirculation. Static haemodynamic measures, such as central venous pressure, have proved unreliable in assessing fluid status [41] but remain the mainstay of resuscitation guidelines [42]. Whilst dynamic tests that employ the interaction between respiratory mechanics and the cardio-circulatory effect of this interaction had been shown to have higher predictive values in predicting fluid responsiveness [43], they require both resources and expertise. In the last decade, inferior vena caval measurement using bedside ultrasound has gained popularity, as it is a non-invasive, dynamic test. A recent meta-analysis of studies that evaluated IVC diameter and variability demonstrated a pooled area under the receiver operating characteristic curve of 0.84 (95 % CI 0.79–0.89) in identifying fluid responsiveness in mechanically ventilated patients [11].

With the widespread acceptability and description of ultrasound to determine fluid responsiveness by IVC and extravascular lung water by lung comets, ultrasound is rapidly becoming the standard of care in ICUs. However, ICUs in many low- to mid-income countries may not have bedside ultrasound available to them [30, 31]. For these resource-limited countries, it is relevant that there should be information for how well bedside ultrasound compares with methods they already have available and may be using on a routine basis. Chest radiographs are commonly done in most units, and over three decades ago, Milne described the vascular pedicle as measured on standard chest radiographs and identified a “normal” value of 48 mm ±5 [44]. Since then, the VPW has been well-described to correlate with chest radiographic signs of pulmonary edema, cardiothoracic ratios, fluid balance, and echo and invasive hemodynamic measurements. Ely et al. in 100 ICU patients described a VPW cutoff value of 70 mm as indicative of volume overload [45]. Martin [46] and Salahuddin et al. [47] demonstrated significant associations with fluid balance; *r* = 0.71, *p* = 0.005 and *r* = 0.88, *p* < 0.001. Thomason et al. [48] and Wichansawakul et al. [49] described significant correlations with pulmonary artery occlusion pressures (PAOP), *r* = 0.45, *p* = 0.0076 and *r* = 0.68, *p* < 0.001, and cardiothoracic ratios on chest radiograph, *r* = 0.49, *p* = 0.0032. Iqbal [50] identified a cutoff VPW value (53 mm) and demonstrated that a left atrial emptying fraction >0.75 had a sensitivity of 74 % and specificity of 94 % for diagnosing raised intravascular volume. Aloizos et al. described a strong correlation (0.785, 0.710, and 0.510) between the VPW and PiCCO-derived parameters: global end-diastolic volume index (GEDI) *p* < 0.001, intrathoracic blood volume (ITBV) *p* < 0.001, and extravascular lung water index (ELWI) *p* < 0.005 [51]. In a meta-analysis of eight studies with 363 subjects, Wang et al. [36] summarized a strong correlation between volume overload (described by heterogeneous methods such as CVP, PAOP, chest radiograph) and VPW, *r* = 0.81 (95 % CI 0.74–0.86). Though this literature provides support for use of the VPW, it does not compare with ultrasound assessment, which arguably may be considered the current standard of care. In this study, we provide one of the first descriptions of VPW with bedside ultrasound.

We were unable to show a correlation between extravascular lung water, as measured by lung comet score, and the VPW. A likely explanation for this is that in critical illness, lung water reflects vascular permeability that itself is not linearly related to increasing hydrostatic pressure (which is what the VPW represents). Patients with sepsis and systemic inflammatory response syndrome may leak fluid into the lung parenchyma and therefore have a high lung comet score but still be intravascularly “dry.” Since a majority of our patients had postoperative major surgery, it is possible that widespread capillary leak was predominant, and therefore, we see this dissociation of the VPW and the lung comet scores.

VPW measurements are simple to perform, and in a retrospective study of 80 ICU patients, Farshidpanah et al. [37] demonstrated that novice physicians-in-training can reliably measure the VPW. Three independent raters performed measurements of VPW. Kappa statistics for inter-rater reliability showed kappa = 0.41, 0.42, and 0.85 for each pair of the three raters.

A limitation of our study is that we included patients who had no anatomic or physiologic dysfunctions that may affect measurements of the vascular pedicle [44, 52], i.e.,valvular heart disease, congestive heart failure, pulmonary hypertension, pericardial disease, and a history of thoracic or cardiac surgery. Patients on high or higher than “physiological” PEEP were excluded since previous investigators have reported that PEEP may affect the IVC measurement and collapsibility index [53, 54]. Another limitation is that most of our patients had a net positive fluid balance and high median pro-BNP values. This may have introduced a selection bias and prevented us from assessing the performance of VPW in volume-depleted patients.

## Conclusions

This study shows a high predictive ability of the VPW for fluid repletion as identified by IVC ultrasound, i.e., a specified value of 64 mm accurately identifies a *fluid replete* state as defined by an IVC diameter greater than 2 cm and loss of IVC variability with the respiratory cycle. Therefore, the VPW may be used confidently to discriminate *fluid repletion* from *fluid responsiveness*. Given the relationship of fluid overload and mortality, these results may assist fluid resuscitation in resource-limited ICUs.

## Availability of supporting data

The dataset supporting the conclusions of this article is included within the article.

## Ethics approval and consent to participate

The study protocol was approved by the institutional research ethics committee (RAC approval no. 2141039), and the study was performed in accordance with the ethical standards laid down in the 1964 Declaration of Helsinki and its later amendments. Informed consent to participate and consent to publish was obtained from the attendant family or designated patient representatives and documented in the medical record.

## Abbreviations

AUC: area under the curve
ICU: intensive care unit
IVC: inferior vena cava
PEEP: positive end-expiratory pressure
ROC: receiver operating characteristic curves
SID: source-image distance
VPW: vascular pedicle width
